# Aortic Thrombosis Associated with Three Types of COVID-19 Vaccine

**DOI:** 10.1155/2023/3562145

**Published:** 2023-10-25

**Authors:** Jose Maria Pereira de Godoy, Fernando Reis Neto, Gabriela Leopoldino da Silva, Henrique Amorim Santos, Henrique Jose Pereira de Godoy

**Affiliations:** ^1^Cardiovascular Surgery Department in Medicine School of Sao Jose do Rio Preto-FAMERP-Brazil, Brazil; ^2^Undergraduate Medicine Course and Stricto-Sensu Postgraduate Course-FAMERP-Brazil, Brazil; ^3^Vascular Surgery Discipline, FAMERP/FUNFARME-Brazil, Brazil; ^4^CNPq (National Council for Research and Development), Brazil

## Abstract

Aortic thrombosis has been studied little in patients with COVID-19 and an association has recently been reported with the vaccine for this disease. The aim of the present study is to report five cases of aortic thrombosis at our institution within a three-month period associated with the COVID-19 vaccine. Five cases of aortic thrombosis were evaluated—three women and two men aged 29, 49, 51, 60, and 79 years. Four thrombi involved the thoracic aortic and one involved the abdominal aorta, with embolisms found in the kidneys, spleen, liver, upper limbs, and lower limbs. Embolectomy was performed on the limbs, and anticoagulant therapy was performed for the abdominal arteries. The patients recovered well and anticoagulant therapy was maintained. Aortic thrombosis is uncommon but was associated with the AstraZeneca vaccine in this case series.

## 1. Introduction

A study reports that, after recovery from the acute phase of COVID-19, patients are at increased risk of a set of cardiovascular problems, such as abnormal heart rhythm, myocardial inflammation, blood clots, stroke, myocardial infarction, and heart failure [1].

Arterial and venous thromboses associated with COVID-19 have been reported in the literature [2–5]. An association has been detected with a state of hypercoagulability, and the risk of developing venous and arterial thromboembolic events is as high as 31% [6]. The Gamma variant has also been described as one of the most thrombogenic variants of COVID-19 [3, 7]. There are also warnings that the adenovirus vector vaccine ChAdOx1 nCoV-19 can result in the rare development of immune thrombotic thrombocytopenia mediated by platelet-activating antibodies against PF4, which clinically mimics autoimmune heparin-induced thrombocytopenia [8, 9].

Aortic thrombosis has been described in case reports of patients with COVID-19 and is associated with important complications [10–12]. One study suggests that the occurrence of the synchronous damage involving both the lumen surface (endothelial dysfunction, endotheliitis, and endothelial detachment) and the adventitia (inflammation and occlusive thrombosis of vasa vasorum) could be the key points related to the fatal outcome in patients with SARS-CoV-2 [13]. Thromboembolic events in unusual locations (thrombi in the aorta with visceral arterial ischemic and in the limbs) are rarely reported in the literature [14].

Aortic thrombosis has been reported little in patients with COVID-19, and an association with COVID-19 vaccines has been found. The aim of the present study was to report five cases of aortic thrombosis at our institution in a three-month period associated with the COVID-19 vaccine.

## 2. Case Series

### 2.1. Case 1

**Figure 1:**
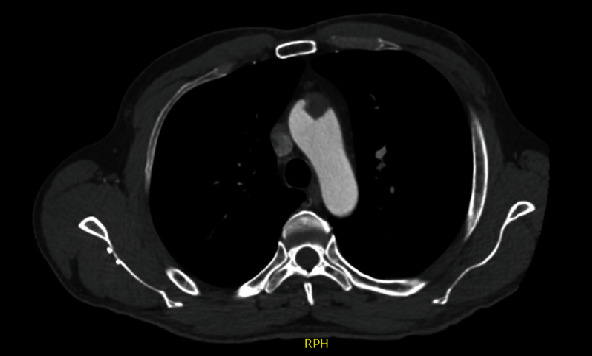
Angiotomography image, axial cut, showing hypodense mural thrombus impacted in aortic arch.

**Figure 2:**
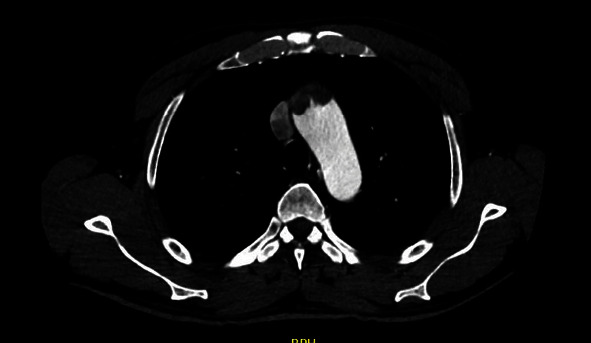
Angiotomography image, axial cut, showing mural thrombi lodged in aortic arch.

**Figure 3:**
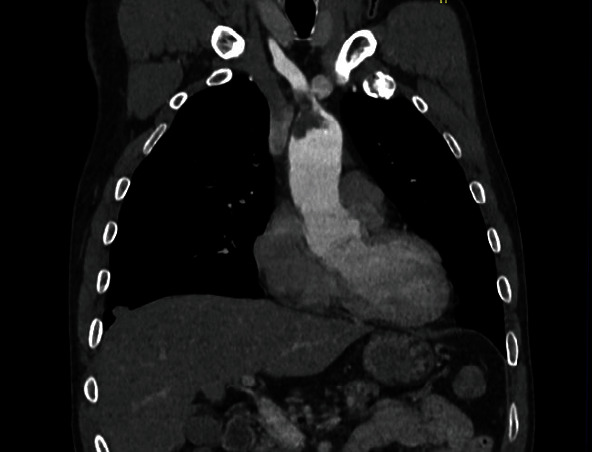
Angiotomography image, coronal cut, showing hypodense thrombi impacted in aortic arch and origin of brachiocephalic trunk.

A forty-nine-year-old male patient with a history of smoking and illicit drug use, having received two doses of the AstraZeneca® vaccine for COVID-19 (first dose in June 2021 and second dose in September 2021) and having tested negative for SARS-CoV-2 infection, was admitted to hospital in October 2021 with pain and paresthesia in the right upper limb. The diagnosis was acute arterial occlusion of the limb due to an embolism in the right ulnar artery, but with the maintenance of a palpable radial pulse. Chest angiotomography revealed hypodense mural thrombi in the aortic arch (Figures 1–3). Conservative treatment was indicated, with antiplatelet aggregation and anticoagulant therapy. The patient was readmitted on January 6, 2022, with pain and paresis of the right upper limb (Rutherford class IIB), presenting only a palpable axillary pulse. The diagnosis was a new embolic event in the right upper limb despite adequate anticoagulation with warfarin. The new angiotomography revealed an increase in the thrombosis of the aortic arch. The patient was submitted to embolectomy of the right upper limb and continued without palpable distal pulses in the limb (only axillary pulse). The embolectomy was performed with a Fogarty catheter, but it was not successful, and the limb was continuous ischemic. Right chest sympathectomy was performed on January 11, 2022, with improvements in paresthesia and pain, and the patient was discharged. Sympathectomy was indicated for pain relief. The patient was readmitted on February 3, 2022, due to infection of trophic lesions in the right upper limb and was submitted to right transhumeral amputation on February 10, 2022. The infection associated with ischemia and pain endangered the patient's life and quality of life, and the decision was made to amputate the limb.

### 2.2. Case 2

**Figure 4:**
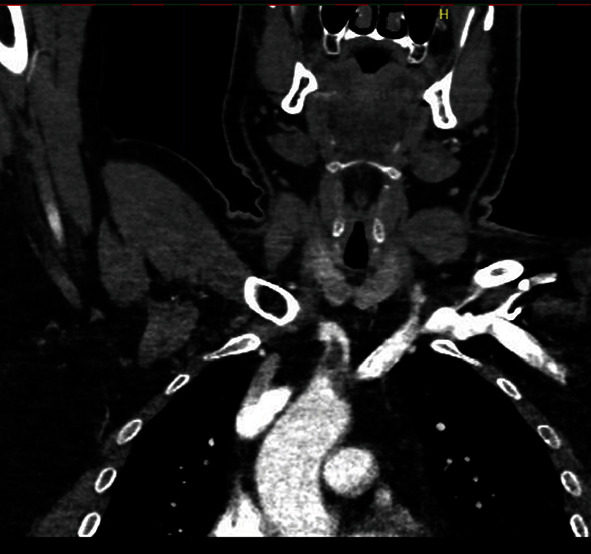
Angiotomography image, coronal cut, showing hypodense thrombi impacted in aortic arch and origin of brachiocephalic trunk.

**Figure 5:**
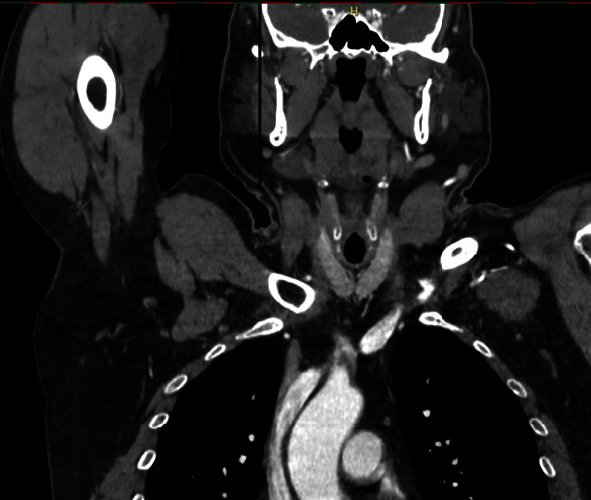
Angiotomography image, coronal cut, showing thrombi in aortic arch and origin of brachiocephalic trunk.

A fifty-one-year-old male patient with a history of smoking and alcohol use, having received two doses of the CoronaVac® vaccine for COVID-19 (first dose in September 2021 and second dose in October 2021) and having tested negative for SARS-CoV-2 infection, was admitted to hospital in January 2022 with pain in the right upper limb and cyanosis of the right first and fifth fingers. The diagnosis was acute arterial occlusion of the right upper limb in the proximal segment of the brachial artery. Chest angiotomography revealed hypodense mural thrombus at the origin of the brachiocephalic trunk (Figures 4 and 5). The patient was submitted to embolectomy with the presence of palpable brachial and ulnar pulses, absence of radial pulse, and improvement in perfusion of the limb. After six days, the patient was submitted to an additional embolectomy of the right upper limb due to a new episode of arterial occlusion, after which the brachial and ulnar pulses were palpable and the radial pulse was absent. New angiotomography revealed a reduction in the dimensions of the thrombus at the origin of the brachiocephalic trunk. The patient received discharge and was counseled to maintain anticoagulation therapy with rivaroxaban, maintaining good perfusion of the limb.

### 2.3. Case 3

**Figure 6:**
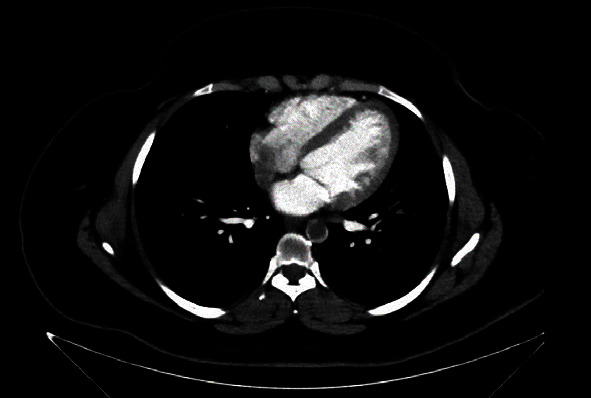
Angiotomography image, axial cut, showing subocclusive thrombus in thoracic aorta.

**Figure 7:**
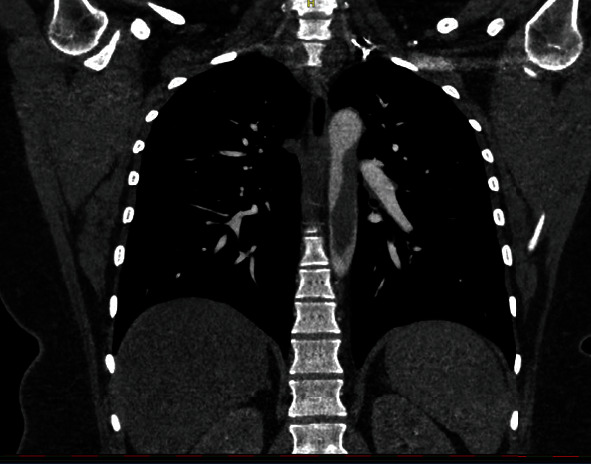
Angiotomography image, coronal cut, showing extensive thrombus in thoracic aorta.

A twenty-nine-year-old female patient with a history of polycystic ovarian syndrome, obesity, smoking, miscarriage, and use of oral anticontraceptive, having received two doses of the AstraZeneca® vaccine for COVID-19 (first dose in June 2021 and second dose in September 2021) and having tested negative for SARS-CoV-2 infection, was admitted to hospital in December 2021 with precordial and epigastric pain as well as pain in the lower limbs at rest. Acute coronary syndrome was discarded. The physical examination revealed only palpable femoral and popliteal pulses; other lower limb pulses were absent, but good perfusion of the limbs was found. Angiotomography revealed a hypodense thrombus in the descending thoracic aorta; signs of embolic infarction in the spleen, liver, and kidneys; and occlusion of the right infragenicular popliteal artery, with segmental arterial occlusions in the right lower limb (Figures 6 and 7). Patency of the left popliteal artery was also found, with segmental occlusions in the arteries of the left lower limb. Conservative anticoagulant treatment was initiated with nonfractionated heparin administered intravenously during hospitalization, resulting in an improvement in pain symptoms. The patient received discharge and was counseled to maintain anticoagulant therapy with warfarin.

### 2.4. Case 4

**Figure 8:**
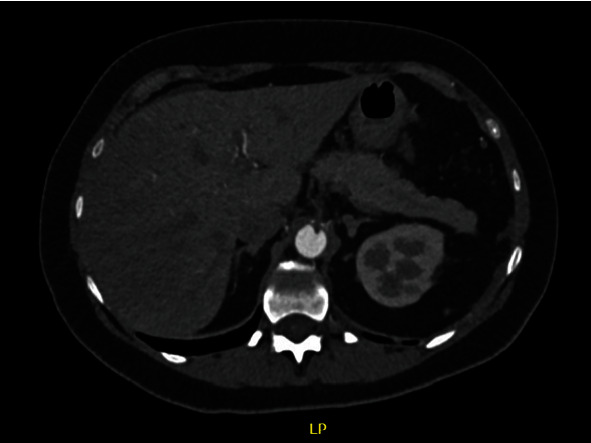
Angiotomography image, axial cut, showing small mural thrombus in abdominal aorta.

**Figure 9:**
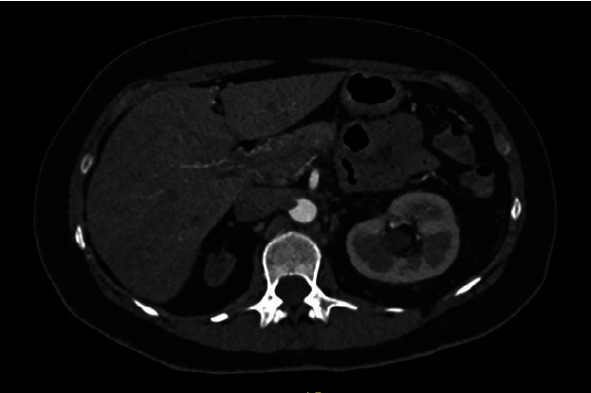
Angiotomography image, axial cut, showing small mural thrombus in abdominal aorta.

A sixty-year-old female patient with a history of nondialytic chronic kidney disease, hypothyroidism, and arterial hypertension and history of infection by SARS-CoV-2 in 2019, having received three vaccines for COVID-19 (first dose with AstraZeneca® in May 2021, second dose with AstraZeneca® in August 2021, and third dose with Pfizer® vaccine in December 2021), was admitted to hospital in February 2022 with diffuse abdominal pain and no signs of peritonitis. Angiotomography revealed hypodense thrombi in the abdominal aorta and thrombosis of the superior mesenteric vein and portal vein, with signs of chronic occlusion of the celiac trunk and no signs of loop ischemia (Figures 8 and 9). Anticoagulant treatment was initiated with nonfractionated heparin administered intravenously during hospitalization, resulting in an improvement in pain symptoms. The patient received discharge and was counseled to maintain anticoagulant therapy with rivaroxaban.

### 2.5. Case 5

**Figure 10:**
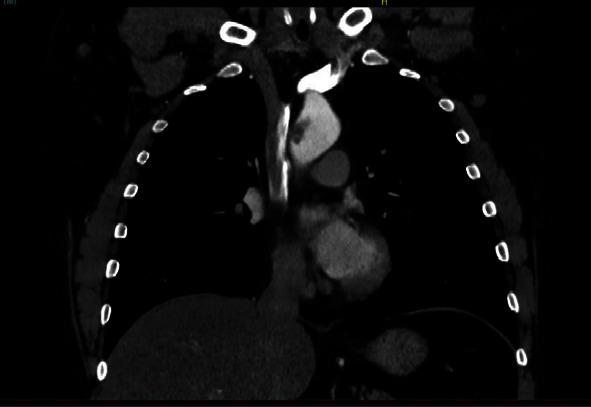
Angiotomography image, coronal cut, hypodense intraluminal thrombus in ascending aorta.

A seventy-nine-year-old male patient with a history of cutaneous porphyria and two episodes of infection by SARS-CoV-2 (first episode in November 2020 and second episode 30 days after being admitted to hospital), having received two doses of AstraZeneca® vaccine for COVID-19, was admitted to hospital with pain and cyanosis in the right fourth finger and subsequently the second and third fingers. Chest and right upper limb angiotomography revealed hypodense, pediculated, amorphous thrombus in the lumen of the aorta at the transition between the ascending segment and proximal bend of the arch as well as signs of occlusion of radial, ulnar, and intermediate arteries (distal third) (Figure 10). Anticoagulant treatment was initiated with nonfractionated heparin administered intravenously during hospitalization, resulting in an improvement in pain and cyanosis. The patient received discharge and was counseled to maintain anticoagulant therapy with rivaroxaban.

This study received approval from the Human Research Ethics Committee of the São José do Rio Preto School of Medicine, SP, Brazil (# 5.647.377).

## 3. Discussion

The present study reports five unusual cases of aortic thrombosis over a three-month period. Vaccine associations were found (three cases with AstraZeneca, one with the Pfizer vaccine, and one with CoronaVac). However, thrombocytopenia was not detected in any of the patients. One of the patients was 29 years old and had a thrombus in the descending thoracic aorta; signs of embolic infarction in the spleen, liver, and kidneys; occlusion of the right infragenicular popliteal artery; and segmental occlusions in the arteries of the right leg, as well as patency of the left popliteal artery and segmental occlusions in the arteries of the left leg. The patient had received two doses of AstraZeneca and had no clinical history of COVID-19 infection, which demonstrates the severity of these cases.

The hospital where these cases were attended is one of the largest reference centers for the treatment of COVID-19 in Brazil, reaching 200 cases in intensive care units/day and also cases in the wards. More than 8000 patients with COVID-19 have been treated in the past two years, with more than 4000 in intensive care units, but only 15 cases of arterial thrombosis have been well documented (including one in an adolescent), three of which were aortic thromboses.[15] This data contrasts with the five cases we had in a three-month period. In the present study, one of the patients tested positive for COVID-19 but had taken two vaccines (two doses of CoronaVac+one dose of AstraZeneca). A strong association was found with the vaccine in all cases. Another important aspect is the involvement of the aortic arch in these patients and in native aortas without evidence of atherosclerosis. We waited eight months after this vaccination period and no new similar cases emerged, therefore an important association with vaccination weeks.

The hypothesis is the occurrence of immunothrombosis involving the involvement of the aorta, mainly the thoracic aorta. Early diagnosis is not always possible; the diagnosis is usually made in the event of an arterial embolic event. The embolic appearance is similar to the usual patterns, for which medical treatment and embolectomy are the primary options. During the pandemic period, most cases of arterial thrombosis in our institution were in the acute phase of COVID-19 infection, demonstrating a direct association with the infectious event. In the present study, the thrombotic event was not associated with virus infection (except in one case), but with a vaccine complication. Thus, prevention of this unpredictable thrombotic event is very difficult, and clinicians should be alerted to atypical vascular conditions in patients who have received the COVID-19 vaccine.

One of the patients returned after treatment for embolism in the upper limb with a new episode of embolism. The new angiotomography revealed an increase in the aortic thrombus. The initial conduct was to discharge the patient with platelet antiaggregation and anticoagulant therapy with aspirin, clopidogrel, and warfarin. Despite this, there was an increase in the thrombus. Another option would be to associate the treatment of the limb or organ affected by the embolism with percutaneous aspiration thrombectomy.

In our hospital, we diagnosed more than 450 cases of deep vein thrombosis associated with COVID-19 and only 15 cases of arterial thrombosis. These thrombotic events increased in frequency with the appearance of the Gamma variant. Thus, there is a difference in the predisposition of thrombotic sites and their prevalence associated with viral variants.

Thrombosis of the thoracic aorta and aortic arch has been observed more frequently in our hospital and associated with vaccines for COVID-19, especially AstraZeneca.

## Data Availability

The data used to support the findings of this study are included within the article.
